# Glucagon-like peptide-1-based therapies and the intersection of metabolic and skin disease

**DOI:** 10.1016/j.jdcr.2026.07.008

**Published:** 2026-07-10

**Authors:** Ana Ormaza-Vera, Clinton W. Enos

**Affiliations:** Department of Dermatology, Eastern Virginia Medical School, Macon & Joan Brock Virginia Health Sciences at Old Dominion University, Norfolk, Virginia

**Keywords:** cardiometabolic disease, GLP-1 receptor agonists, hidradenitis suppurativa, immunometabolism, psoriasis, systemic inflammation

*To the Editor*: The development of the glucagon-like peptide-1 (GLP-1) receptor agonists (GLP-1RAs) and the dual glucose-dependent insulinotropic polypeptide/GLP-1RAs is revolutionizing the management of metabolic disease. Perhaps most impressive is that their clinical significance extends far beyond their approved indications and touches seemingly every organ system, even the skin.

GLP-1RAs are incretin-based drugs that enhance glucose-dependent insulin secretion, suppress glucagon release, delay gastric emptying, and promote satiety, resulting in substantial improvements in glycemic control, weight reduction, and favorable metabolic effects.^1^^,^^2^ The metabolic state of an individual can have a broad impact on cellular, tissue, and systemic processes, both indirectly by regulating nutrient availability and directly by delivering signaling metabolites. The interrelation of inflammation and insulin resistance is a prime example. Because these same inflammatory and metabolic pathways are central to the pathogenesis of many dermatologic conditions, this interplay supports the ever-growing recognition that inflammatory skin diseases are best understood as systemic disorders, often associated with cardiometabolic comorbidities and an increased risk of future cardiovascular events.^3^, ^4^, ^5^, ^6^, ^7^, ^8^, ^9^ As such, the pleiotropic effects of GLP-1–based therapies may be directly relevant to inflammatory skin disease.

In fact, clinical studies have demonstrated reductions in systemic inflammatory markers, including C-reactive protein, interleukin 6, and tumor necrosis factor-α, in patients treated with GLP-1RAs, with these effects thought to be partially independent of weight loss.^1^^,^^10^, ^11^, ^12^ These pathways intersect with established mechanisms implicated in psoriasis and hidradenitis suppurativa, providing a biologically plausible rationale for their investigation as adjunctive therapies.

Evidence from open-label, single-center studies suggests that GLP-1–based therapies may result in clinically meaningful improvements in body weight, metabolic parameters, inflammatory markers, and disease activity in patients with hidradenitis suppurativa having concomitant obesity.^13^^,^^14^ Efforts are also well underway in psoriasis and psoriatic arthritis. The phase 3b TOGETHER PsO^15^ and TOGETHER PsA^16^ studies evaluated tirzepatide in combination with ixekizumab and have reported superiority over monotherapy with ixekizumab.^17^^,^^18^ While current phase 4 studies aim to assess the addition of glucose-dependent insulinotropic polypeptide/GLP-1RAs in clinical practice among patients with psoriasis treated with ixekizumab,^19^^,^^20^ investigator-initiated studies, such as SEMAPSO^21^ and SEMPSO,^22^ are assessing the effects of GLP-1RAs on both cutaneous outcomes and metabolic inflammation in patients with moderate-to-severe psoriasis and obesity. These studies not only reflect the growing interest in integrated treatment approaches that address metabolic comorbidity alongside immune–mediated skin disease but also the potential mechanisms intertwining them.

GLP-1–based therapies represent an exciting therapeutic approach for managing conditions that have both metabolic and inflammatory components. As highlighted in this virtual issue, emerging case reports and small clinical studies underscore both the therapeutic potential and the evolving dermatologic safety profile of GLP-1–based agents.^23^, ^24^, ^25^, ^26^, ^27^, ^28^, ^29^, ^30^, ^31^, ^32^, ^33^, ^34^, ^35^, ^36^, ^37^, ^38^, ^39^, ^40^ As our experience grows with these therapies, questions persist regarding optimal patient selection, durability of dermatologic response, independence from weight loss, integration with established biologic and small molecule therapies, and long-term safety. Addressing these gaps will require well-designed prospective trials focused on dermatologic endpoints, complemented by real-world data and carefully documented clinical observations.

## Conflicts of interest

None disclosed.
